# Supplementary material for: Complexome profiling of the Chlamydomonas psb28 mutant reveals TEF5 as an early PSII assembly factor
Source: Plant Cell. 2025 Mar 17;37(6):koaf055. doi: 10.1093/plcell/koaf055 (PMC12164590; doi:10.1093/plcell/koaf055)
Supplement: koaf055_Supplementary_Data [file koaf055_supplementary_data.zip › psb28_interactive.zip!/plots/intensity/qualityPlots/peptides/LPA1_Cre10.g430150.t1.2.html]

### Cre10.g430150.t1.2

Trivial name: LPA1  
SeqCoverage [median]: 27.20  
Mass: 49.55 kDa  
  
 

| Sequence | MissedCleavage | Mass [Da] | wtIntensity | muIntensity | Score |
| --- | --- | --- | --- | --- | --- |
| AIKEAEGQYLNLR | 1 | 1503.80 | 371.96 | 496.80 | 93.62 |
| AQAVELDPEDKLR | 1 | 1482.77 | 8371.24 | 10331.55 | 75.48 |
| AVSVVPIIFEK | 0 | 1200.71 | 1223.70 | 0.00 | 80.24 |
| EAEGQYLNLR | 0 | 1191.59 | 0.00 | 1056.17 | 108.46 |
| FSEAVEAIER | 0 | 1149.57 | 290.25 | 2403.83 | 125.97 |
| GQVRPVIVAGSR | 1 | 1237.73 | 1024.85 | 2788.44 | 72.29 |
| GTEVFKNQNNLDEAVR | 1 | 1832.90 | 662.55 | 764.69 | 61.10 |
| LIVALRDDDLR | 1 | 1297.74 | 1021.17 | 7254.73 | 59.43 |
| NQNNLDEAVR | 0 | 1171.56 | 933.45 | 1369.66 | 90.60 |
| SQQYQPTSAR | 0 | 1164.55 | 1274.99 | 1057.88 | 125.48 |
| VGLEMQPNDDEAR | 0 | 1472.66 | 2090.61 | 1400.44 | 99.75 |
| VSVLGGLVEADKK | 1 | 1313.76 | 1130.60 | 2757.39 | 54.65 |
|  |  |  | 18395.4 | 31681.6 |  |
